# Electroacupuncture activates corticotrophin-releasing hormone-containing neurons in the paraventricular nucleus of the hypothalammus to alleviate edema in a rat model of inflammation

**DOI:** 10.1186/1472-6882-8-20

**Published:** 2008-05-12

**Authors:** Aihui Li, Lixing Lao, Yi Wang, Jiajia Xin, Ke Ren, Brian M Berman, Ming Tan, Ruixin Zhang

**Affiliations:** 1 Center for Integrative Medicine, School of Medicine, University of Maryland, Baltimore, MD 21201, USA; 2 Shanghai University of Traditional Chinese Medicine, Yueyang Affiliated Hospital, Shanghai, China; 3 Dept. of Biomedical Sciences, Dental School, University of Maryland, Baltimore, MD 21201, USA; 4 Division of Biostatistics, University of Maryland Greenebaum Cancer Center, Baltimore, MD 21201, USA

## Abstract

**Background:**

Studies show that electroacupuncture (EA) has beneficial effects in patients with inflammatory diseases. This study investigated the mechanisms of EA anti-inflammation, using a rat model of complete Freund's adjuvant (CFA)-induced hind paw inflammation and hyperalgesia.

**Design:**

Four experiments were conducted on male Sprague-Dawley rats (n = 6–7/per group). Inflammation was induced by injecting CFA into the plantar surface of one hind paw. Experiment 1 examined whether EA increases plasma adrenocorticotropic hormone (ACTH) levels. Experiments 2 and 3 studied the effects of the ACTH and corticotropin-releasing hormone (CRH) receptor antagonists, ACTH_(11–24) _and astressin, on the EA anti-edema. Experiment 4 determined whether EA activates CRH neurons in the paraventricular nucleus of the hypothalammus. EA treatment, 10 Hz at 3 mA and 0.1 ms pulse width, was given twice for 20 min each, once immediately post and again 2 hr post-CFA. Plasma ACTH levels, paw thickness, and paw withdrawal latency to a noxious thermal stimulus were measured 2 h and 5 h after the CFA.

**Results:**

EA significantly increased ACTH levels 5 h (2 folds) after CFA compared to sham EA control, but EA alone in naive rats and CFA alone did not induce significant increases in ACTH. ACTH_(11–24) _and astressin blocked EA anti-edema but not EA anti-hyperalgesia. EA induced phosphorylation of NR1, an essential subunit of the N-methyl-D-aspartic acid (NMDA) receptor, in CRH-containing neurons of the paraventricular nucleus.

**Conclusion:**

The data demonstrate that EA activates CRH neurons to significantly increase plasma ACTH levels and suppress edema through CRH and ACTH receptors in a rat model of inflammation.

## 1. Background

Clinical trials show that electroacupuncture (EA) has beneficial effects in patients with various inflammatory diseases [1]. Studies demonstrate that EA significantly inhibits complete Freund's adjuvant (CFA)-induced hind paw inflammation and hyperalgesia in a rat model [2,3]. However, the underlying mechanisms of acupuncture are still not completely understood.

We recently demonstrated that EA significantly increased plasma corticosterone levels in rats with hind paw inflammation compared to sham EA control. Adrenalectomy blocked EA-produced anti-edema, but not EA anti-hyperalgesia. RU486, a prototypical glucocorticoid receptor antagonist, also prevented EA anti-edema. These data suggest that adrenal gland-secreted corticosterone mediates EA anti-edema [2,4].

Previous study suggests that the paraventricular nucleus (PVN) of the hypothalamus is involved in acupuncture analgesia. Electrical stimulation of the PVN significantly increased the pain threshold and enhanced acupuncture analgesia [5]. Pituitary gland involvement in EA has been inconclusive. It was reported that hypophysectomy (HYPOX) attenuated acupuncture analgesia in mice [6] and rats [7,8], and that inhibition of the pituitary function by dexamethasone treatment reduced acupuncture analgesia [9]. These data indicate the involvement of the pituitary gland in EA analgesia. However, it was also reported that HYPOX did not alter EA-induced inhibition of the writhing response in mice [10] and that the pituitary gland was not involved in production of EA analgesia in rats [11]. These discrepancies are likely related to the status of the animals (e.g. length of time after surgery) and to the tests used in the studies. All of these studies were conducted on animals with mechanical or chemical pituitary gland damage and transient noxious stimuli. Corticotropin-releasing factor (CRH) and adrenocorticotropic hormone (ACTH) are secreted by the PVN and pituitary gland, respectively, and drive the secretion of corticosterone from adrenal glands. Whether they are involved in EA-produced anti-edema and anti-hyperalgesia has not been previously investigated using their respective antagonists in an inflammatory pain animal model. Because uninjured models do not mimic the chronic pathological conditions seen clinically, we used a CFA-inflamed rat model to test the hypothesis that EA activates CRH-containing neurons in the PVN and increases plasma ACTH levels to ameliorate inflammation and hyperalgesia.

## 2. Methods

### 2.1 Animal preparation

Male Sprague-Dawley rats weighing 280–320 g (Harlan, Indianapolis, IN) were kept under controlled conditions (22°C, relative humidity 40%–60%, 12-hour alternate light-dark cycles, food and water *ad libitum*). The animal protocols were approved by the Institutional Animal Care and Use Committee (IACUC) of the University of Maryland School of Medicine.

### 2.2 Experimental design

Four experiments were conducted. In Experiment 1 we measured plasma ACTH levels to examine whether EA regulates ACTH secretion. Rats were divided into four groups (n = 6 per group): CFA (0.08 ml) + EA, CFA + sham EA, CFA + no treatment, and naive + EA. EA was administered at 10 Hz, 3 mA, 0.1 ms pulse width for two 20-min periods, once at the beginning and once at the end of a 2-h period starting immediately after CFA injection. Blood samples (0.5 ml) were taken from each rat at baseline (before inflammation and/or EA) and 2 h and 5 h after inflammation. In Experiment 2, an ACTH antagonist, ACTH_(11–24) _(Bachem), was used to study the effects of ACTH on the therapeutic actions of EA. Rats were divided into four groups (n = 7 per group): 1) CFA (0.06 ml) + Vehicle + Sham EA, 2) CFA + Vehicle + EA, 3) CFA + ACTH_(11–24) _+ Sham EA and 4) CFA + ACTH_(11–24) _+ EA. A 2 mg/ml concentration of ACTH_(11–24) _was dissolved in saline, and 2 mg/kg of ACTH_(11–24) _or vehicle was intravenously (i.v.) injected 5 min before each of two 10 Hz EA treatments. The degree of edema, indicative of the intensity of inflammation, was quantified by measuring paw thickness with a Laser Sensor (AR200-50, Acuity, Portland, OR) 2 h and 5 h after a CFA injection into a hind paw. The paw withdrawal latency (PWL) test was conducted at the same time points. The investigator who conducted the measurement was blinded to the treatment assignments. Experiment 3 was to determine whether a CRH receptor antagonist, astressin, influences EA action. CFA-inflamed rats were divided into four groups (n = 7 per group): 1) CFA + Vehicle + Sham EA, 2) CFA + Vehicle + EA, 3) CFA + Astressin (Sigma) + Sham EA and 4) CFA + Astressin + EA. The paw thickness was measured with the same Laser Sensor. A 2 mg/ml concentration of astressin was dissolved in saline, and 0.2 mg/kg of astressin or vehicle was injected (i.v.) 5 min before each of two 10 Hz EA treatments. Experiment 4 was to determine whether EA activates CRH-containing neurons in the PVN. This was done by demonstrating double-labeling of CRH and phosphorylated NR1, an essential subunit of the N-methyl-D-aspartic acid (NMDA) receptor, the phosphorylation of which is known to modulate NMDA receptor activity and can be used as an indicator of NMDA receptor activation. To avoid the effect of CFA-induced inflammation on the PVN neurons, 3 naive rats given EA treatment and 2 untreated naive rats were used in this experiment.

### 2.3 Intravenous cannulation and blood sample collection

For intravenous cannulation, animals were anesthetized with sodium pentobarbital (50 mg/kg) intraperitoneally (i.p.) and surgically implanted with a subcutaneous jugular catheter (Braintree Scientific, Inc). The catheter was secured with Mersilene surgical mesh (General Medical, New Haven, CT) at the jugular vein. It exited at the animal's back through a 22-gauge tubing secured with mesh. Antibiotic ointment was applied to the wound. To prevent clogging, catheters were flushed every third day with 0.15 ml of gentamicin (120 μg/ml). At baseline and 2 h and 5 h post-CFA, 0.5 ml of blood was withdrawn, and the lost volume was replaced by an equal volume of saline. Blood was centrifuged (1310 g) for 15 min at 4°C. The plasma was collected and stored at -80°C until assayed. ACTH levels were measured with a commercially available ELISA kit (MD Biosciences, Inc.) using the procedure recommended by the manufacturer. The detection limit of the kit is 0.46 pg/ml. The antibody in the kit specifically reacts with ACTH and has less than 1% cross-reactivity with alpha-MSH (Melanocyte-stimulating hormone) and beta-endorphin. ACTH concentrations (pg/ml) were determined by comparing samples to a standard curve generated with the kit.

### 2.4 Inflammation and hyperalgesia testing

Inflammation was induced by subcutaneously injecting CFA (0.5 mg/ml heat-killed *Mycobacterium tuberculosis *suspended in an 1:1 oil/saline emulsion; Sigma, St. Louis, MO) into the plantar surface of one hind paw of each rat using a 25-gauge hypodermal needle [12]. Inflammation appeared within 2 h of the injection and peaked between 6–24 h. PWL was tested with a previously described method [12,13]. Each rat was placed under an inverted clear plastic chamber on the glass surface of the Paw Thermal Stimulator System (UCSD, San Diego) and allowed to acclimatize for 30 min before the test. A radiant heat stimulus was applied to the plantar surface of each hind paw from underneath the glass floor with a projector lamp bulb (CXL/CXR, 8 V, 50 W). PWL to the nearest 0.1 sec was automatically recorded when the rat withdrew its paw from the stimulus. Stimulus intensity was adjusted to derive a baseline PWL of approximately 10.0 s in naive animals. Paws were alternated randomly to preclude "order" effects. A 20-sec cut-off was used to prevent tissue damage. Four tests were conducted, with a 5-min interval between each test. Mean PWL was established by averaging the tests.

### 2.5 Acupuncture Treatment Procedures

To maximize the anti-inflammatory effect and to treat animals prophylactically, the EA treatment was given twice, for 20 min each, once immediately after the administration of CFA and again 2 h post-CFA [3]. EA parameters of 10 Hz, 3 mA, 0.1 ms pulse width, which showed significant anti-inflammatory and anti-hyperalgesic effects on the rat inflammation model in our previous studies [2,3], were used in the present study.

The equivalent of the human acupoint Huantiao (GB30) was chosen for bilateral needling based on traditional Chinese medicine (TCM) meridian theory [14], on its successful use in our previous studies, and on studies by others [3,15,16]. Based on our previous point-specificity study [3], EA produced better anti-hyperalgesia at GB30 than at acupoint Waiguan (the fifth acupoint on the Triple Energizer Meridian, TE 5) on the forepaw or at two non-specific points, an abdominal point and a point on the quadriceps opposite to GB30. Waiguan is located dorsally between the radius and ulna, two units (based on the standard acupuncture measurement of twelve units between the transverse cubital crease and the transverse wrist crease) above the transverse crease of the wrist. Underneath are the posterior interosseous nerve and the anterior interosseous nerve. In humans, GB30 is located at the junction of the lateral 1/3 and medial 2/3 of the distance between the greater trochanter and the hiatus of the sacrum; underneath are the sciatic nerve, inferior gluteal nerve and gluteal muscles [17]. GB30 was located on the rat's hind limbs using the comparable anatomical landmarks. After cleaning the skin with alcohol swabs, a disposable acupuncture needle (0.25 mm thickness × 0.5 inch length) was inserted obliquely approximately 0.5 inch deep at GB30 on each of the animal's hind limbs, and a pair of electrodes was attached to the handles of the needles. The needles and electrodes were stabilized with adhesive tape.

EA stimulation was delivered by an electrical stimulator (A300 Pulsemaster, World Precision Instruments) via an isolator (A360D Stimulus Isolator, World Precision Instruments) that delivers steady direct current. This bilateral, cross-limb connection has been used previously by our team and others with no adverse effects [3,18]. Similar connections are frequently used in clinic, and no adverse effects have been reported [19,20].

While the EA frequency was held constant, intensity was adjusted slowly over the period of approximately 2 min to the designated level of 3 mA, which is the maximum EA current intensity that a conscious animal can tolerate [3]. Mild muscle twitching was observed. During EA treatment, each rat was placed under an inverted clear plastic chamber (approximately 5 × 8 × 11 inches) but was neither restrained nor given anesthetic. The animals remained awake and still during treatment and showed no observable signs of distress. For sham treatment control, acupuncture needles were inserted bilaterally into GB30 without electrical stimulation or manual needle manipulation. This procedure produced no anti-hyperalgesic effect on this animal model in our previous study [3]. Since it is comparable to the treatment procedure but lacks therapeutic effect, we used it as sham control in this study.

### 2.6 Immunohistochemistry

Rats in experiment 4 were deeply anesthetized after EA treatment with sodium pentobarbital (60 mg/kg, i.p.) and immediately perfused transcardially with 4% paraformaldehyde (Sigma) in 0.1 M phosphate buffer (PB) at pH 7.4. The hypothalamus was removed, immersed in the same fixative for 2 h at 4°C, and transferred to 30% sucrose (w/v) in PB saline (PBS) overnight for cryoprotection. Forty micron-thick sections were cut on a cryostat and were double-stained for p-NR1 and CRH using the immunofluorescence method. Sections were blocked in PBS with 10% normal donkey serum for 60 min, incubated overnight at room temperature with a mixture of goat polyclonal antibody against CRH (Santa Cruz Biotechnology, Inc., 1:100) and rabbit polyclonal antibody against P-NR1 (Upstate, Serine 896, 1:100). After three 10-minute washings in PBS, sections were incubated in a mixture of CY2-conjugated donkey anti-rabbit (1:100, Jackson ImmunoResearch Laboratories) and CY3-congugated donkey anti-goat (1:100) for 1 h at room temperature. Control sections were similarly processed, except that the primary antisera were omitted. The stained sections were mounted on gelatin-coated slides, coverslipped with aqueous mounting medium (Biomeda Corp., CA) and examined under a Nikon fluorescence microscope. Control sections without primary antiserum showed no immunoreactive staining.

### 2.7 Data Analysis

For clarity, plasma ACTH level data are presented as percent changes of the baseline level: [(level_2–5 h_-level_baseline_)/level_baseline_] × 100%. Paw thickness data were presented as mean ± SEM. PWL data are presented as mean ± SEM or (PWL_2–5 h _- PWL_baseline_)/PWL_baseline _× 100%. Data were analyzed using analysis of variance (ANOVA) with repeated measures followed by *post-hoc *Tukey's multiple comparisons (GraphPad InStat). P < 0.05 was set as the level of statistical significance.

## 3. Results

### 3.1 EA increased plasma levels of ACTH in inflamed rats

**Figure 1:**
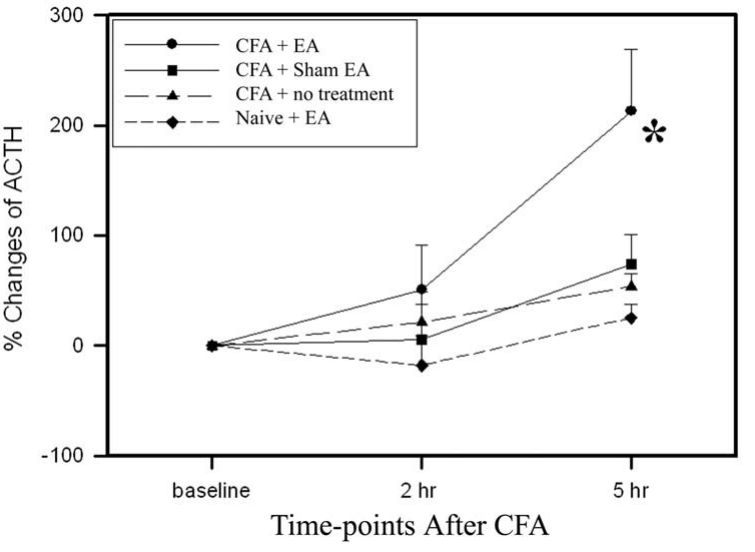
**Effects of EA treatment on plasma ACTH levels (% changes vs baseline, n = 6 per group, mean ± SEM).** EA treatment induced no significant changes of plasma ACTH in uninflamed rats. CFA-induced inflammation alone resulted in no significant changes in plasma ACTH levels. EA treatment in inflamed rats significantly increased ACTH levels compared to sham EA. *P < 0.05 compared to sham EA.

As shown in Figure 1, the plasma ACTH levels of the EA-treated inflamed rats were significantly higher 5 h post-CFA injection than those of sham-treated inflamed rats. The plasma ACTH levels of sham-treated inflamed rats did not show significant change from baseline during the same period. In contrast to CFA-inflamed rats, EA treatment of uninflamed rats produced no significant changes in plasma ACTH level. CFA-induced inflammation alone did not cause significant plasma ACTH changes.

### 3.2 ACTH (11–24) blocked EA anti-edema but not EA anti-hyperalgesia

**Figure 2:**
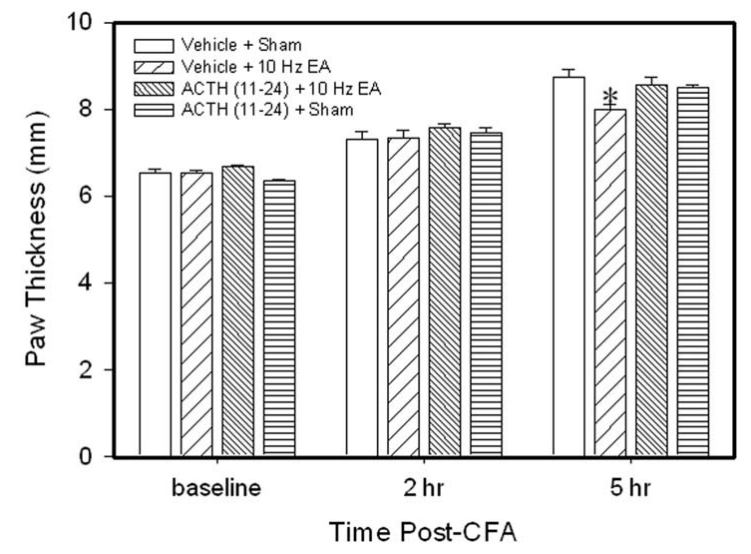
**Effects of ACTH_(11–24)_, an ACTH receptor antagonist, on EA-produced inhibition of edema (n = 7 per group, mean ± SEM).** All rats were CFA-inflamed. Note that ACTH_(11–24) _prevented EA-produced inhibition of edema 5 h post CFA. Edema was determined by increased paw thickness (mm). * P < 0.05 compared to vehicle plus sham EA.

We previously reported that EA significantly inhibits edema compared to sham EA control in CFA-injected rats [2,4]. Figure 2 shows that paw thickness in rats with EA plus vehicle was significantly less than that in rats with sham EA plus vehicle 5 hr post-CFA injection, confirming that EA significantly inhibited edema compared to sham control. Paw thickness in rats with sham EA plus ACTH (11–24) was not different from that in rats with sham EA plus vehicle, indicating that ACTH (11–24) had little effect on edema. However, paw thickness in EA-treated rats plus ACTH (11–24) was not different from that in sham EA rats plus vehicle, indicating that ACTH (11–24) blocked EA anti-edema.

**Figure 3:**
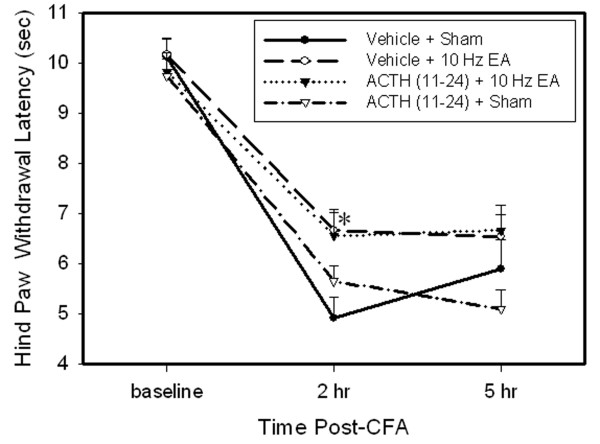
**Effects of ACTH_(11–24) _on EA-produced inhibition of hyperalgesia (n = 7 per group, mean ± SEM).** Note that EA significantly inhibited hyperalgesia 2 h post-CFA, and ACTH_(11–24) _did not prevent EA-produced inhibition of hyperalgesia. * P < 0.05 compared to vehicle plus sham EA.

Figure 3 shows the effects of ACTH_(11–24) _on EA-produced anti-hyperalgesia. Before CFA, overall mean baseline PWL to noxious heat stimuli was similar in all groups of rats (from 9.81 to 10.17 sec), and there was no significant difference in PWLs between left and right hind paws. Following injection of CFA, PWL of the injected paw was significantly less than that of the contralateral hind paw, which was unchanged from baseline (data not shown). EA treatment significantly (P < 0.05) increased PWL of the injected paw 2 h post-CFA compared to sham EA, indicating that EA inhibited hyperalgesia. ACTH (11–24) pretreatment did not affect EA anti-hyperalgesia in EA-treated rats and showed no effect on hyperalgesia in control rats with sham EA.

### 3.3 Astressin blocked EA anti-edema but not EA anti-hyperalgesia

**Figure 4:**
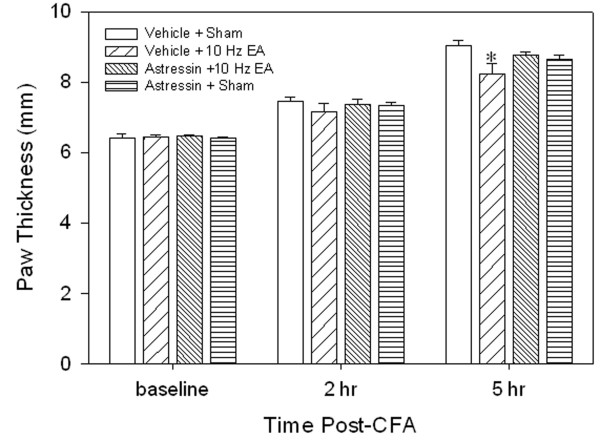
**Effects of astressin, a CRH receptor antagonist, on EA-produced inhibition of edema (n = 7 per group, mean ± SEM).** All rats were CFA-inflamed. Note that astressin prevented EA-produced inhibition of edema 5 hr post CFA. * P < 0.05 compared to vehicle plus sham EA.

Figure 4 shows that EA plus vehicle significantly decreased paw thickness compared to sham EA plus vehicle, indicating that EA inhibited edema. However, EA plus astressin did not significantly alleviate hind paw edema compared with the sham EA rats plus vehicle, indicating that astressin blocked the EA-produced anti-edema effect. Sham EA plus astressin did not significantly change hind paw edema compared with that of sham EA rats plus vehicle, indicating that astressin had little effect on edema.

**Figure 5:**
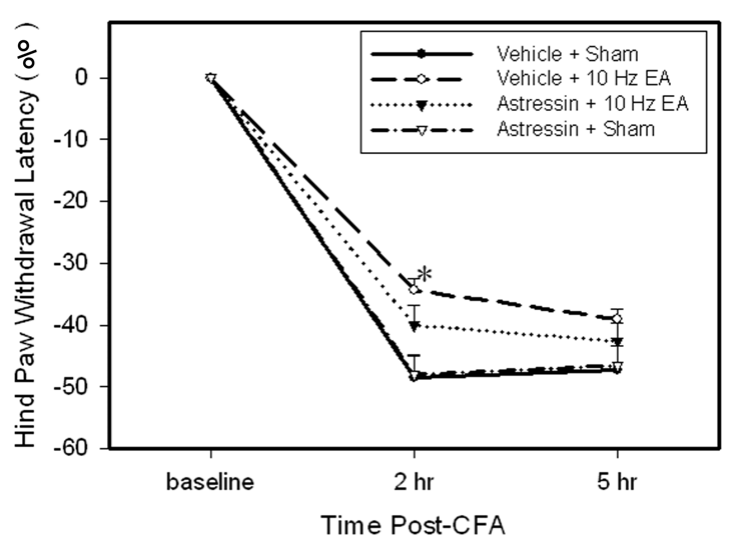
**Effects of astressin on EA-produced inhibition of hyperalgesia (n = 7 per group, mean ± SEM).** Note that EA significantly inhibited hyperalgesia 2 h post-CFA, and astressin partially prevented EA-produced inhibition of hyperalgesia. * P < 0.05 compared to vehicle plus sham EA.

Figure 5 shows the effects of astressin on EA-produced anti-hyperalgesia. PWL of the injected paw was significantly shorter compared to baseline while contralateral PWL did not change (data not shown). EA treatment significantly (P < 0.05) increased PWL of the injected paw 2 h post-CFA compared to sham EA, demonstrating that EA inhibited hyperalgesia. Astressin pretreatment partially inhibited EA anti-hyperalgesia but had no effect on hyperalgesia in sham control rats.

### 3.4 EA activated CRH-containing neurons in the PVN

**Figure 6:**
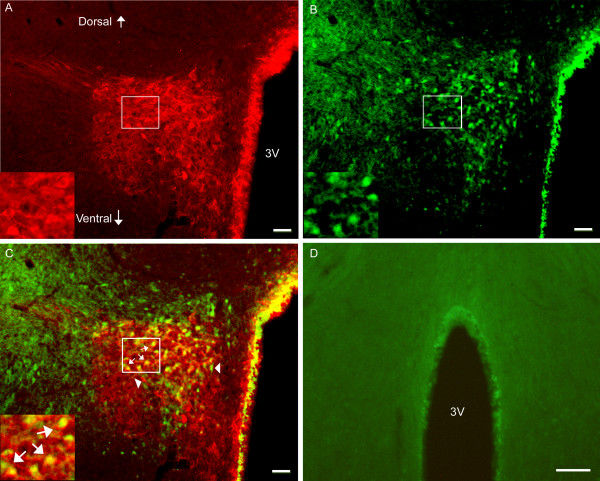
**Photomicrographs showing co-localization of CRH and p-NR1 in the PVN.** A-C: Sections from EA-treated naive rats were double-labeled with anti-CRH (red) and anti-p-NR1 (green). A: CRH-immunoreactive neurons in the PVN. B: p-NRI-immunoreactive neurons in the PVN. C: Merged graphs of A and B. Small arrows indicate examples of double-labeled CRH/p-NR1 neuron (yellow); Arrowheads point to single-labeled CRH and p-NR1. The insets in A, B and C are higher magnification of the square areas in A, B and C, respectively. D: Sections from untreated naive rats were signle-labeled with anti-p-NR1 and showed no labeling of P-NR1. Scale bars represent 50 μm in A, B, C, and 250 μm in D.

Double immunofluorescence labeling showed that EA induced phosphorylation of NR1 subunit of the NMDA receptor in the PVN. Seventy-three percent of phosphorylated NR1 staining was localized in the CRH-containing neurons. This indicates that EA mainly activated CRH-containing neurons in the PVN. Some neurons were single-labeled with CRH, suggesting that they were not activated by EA (Figure 6). Untreated naive rats did not show NR1 phosphorylation in the PVN. See Figure 6.

## 4. Discussion

The present study found that EA activates CRH-containing neurons in the PVN. The PVN is a pivotal nucleus that responds to various stimuli including stress [21]. However, as discussed below, the EA procedures used in this study have little stress effect. Thus, the neuron activation in the PVN of EA-treated rats is considered a specific response to EA, not a general stress response. Previous study also demonstrates that EA significantly activates the PVN [22]. Our data further show that EA-activated neurons in the PVN contain CRH, which indicates that EA treatment may induce CRH secretion. The secreted CRH acted on CRH receptors to increase ACTH release, which is supported by the data showing that EA significantly increases plasma ACTH levels in CFA-inflamed rats compared to sham EA. These data are consistent with our recent report that EA increases endogenous corticosterone secretion [4]. The secreted CRH and ACTH activated their respective receptors, leading to the inhibition of edema. This is evidenced by our experiments on CRH and ACTH antagonists. Pretreatment with a CRH antagonists, astressin and an ACTH antagonist, ACTH_(11–24)_, prevented EA anti-edema. Taken together, these results suggest that in pathological conditions EA activates the HPA axis to increase CRH, ACTH, and glucocorticoid secretion and suppress inflammatory responses.

It should be mentioned that EA significantly increased plasma ACTH levels in inflamed rats but not in naive rats, which is consistent with our previous report that EA increased plasma corticosterone levels in inflamed rats but did not affect those of naive rats [4]. It is well known that stress induces higher ACTH plasma levels. So since rats with persistent inflammation respond to external stimuli differently than do healthy rats [23], it is possible that although EA does not produce stress in naive rats it may induce stress in inflamed rats. However, our previous study demonstrated that the same stimulation intensity at acupoint GB30 as that used in this study did not significantly change heart rate or blood pressure, both of which are indicators of stress response, in CFA-inflamed animals [2]. This suggests that the stress level, if any, induced by such EA treatment is minimal in inflamed rats. Given these data, the ACTH level increase in EA-treated CFA rats was mainly induced by EA treatment and stress caused little, if any, of the increase. Moreover, the discrepancy between inflamed and naive rats in response to the same EA treatment suggests that EA affects healthy and pathological conditions differently. This is supported by a previous study reporting that naloxone completely blocked EA analgesia in healthy rats but partly blocked EA anti-hyperalgesia in carrageenan-induced inflammatory rats [23]. Collectively, these studies suggest that EA works differently in healthy and pathological conditions.

It is noted that astressin partially blocked EA anti-hyperalgesia, suggesting that endogenous CRH play a little part in EA anti-hyperalgesia. It has been well documented that EA induces higher plasma beta-endorphin levels and that beta-endorphin is involved in pain modulation [24,25]. So, it is hypothesized that EA activates CRH neurons in the PVN to secret CRH, which in turn stimulates the release of beta-endorphin to inhibit pain. A previous study using direct stimulation of the PVN showed that the PVN was involved in acupuncture analgesia and that intracerebral pretreatment with antiserum against vasopressin, which is secreted mainly by PVN, attenuated the effect of acupuncture analgesia [5]. It suggests that PVN-produced vasopressin may be involved in acupuncture analgesia. Taken together, these studies suggest that EA action involves a variety of PVN chemicals.

It is also noticed that ACTH_(11–24) _did not block EA anti-hyperalgesia, suggesting that endogenous ACTH plays little role in EA anti-hyperalgesia. Previous studies using HYPOX showed that the pituitary gland was involved in acupuncture analgesia [7,8]. The discrepancy between previous studies and ours may be due to the difference in animal models, inflammatory vs. uninflamed animals, since EA affects healthy and pathological conditions differently, as stated above. Another difference is that we used antagonists in our study while the previous studies used HYPOX that took away all pituitary-secreted molecules, including beta-endorphin. Logically, less involvement of CRH and no involvement of ACTH in EA anti-hyperalgesia suggest that EA may produce anti-hyperalgesia by affecting the nervous system. Our recent studies [26] demonstrate that EA activates brainstem nuclei, which are involved in descending inhibitory modulation of spinal transmission of noxious messages, to inhibit the transmission of noxious messages at the spinal cord. Our previous studies demonstrate that spinal mu and delta opioid systems are involved in EA anti-hyperalgesia in the same inflammatory pain rat model [12].

## 5. Conclusion

The present study demonstrates that EA activates CRH-containing neurons to significantly increase plasma ACTH levels and suppress edema through CRH and ACTH receptors in a rat model of inflammation. EA-produced inhibition of edema involves the activation of NMDA receptors in CRH-containing neurons of the paraventricular nucleus.

## Competing interests

The authors declare that they have no competing interests.

## Authors' contributions

RZ and AL designed the study, carried out the animal surgery, acupuncture treatment and blood collections, and drafted the manuscript. YW and JX carried out the behavioral tests. KR, BMB and LL participated in the design and helped draft the manuscript. MT performed the statistical analysis. All authors read and approved the final manuscript.

## Pre-publication history

The pre-publication history for this paper can be accessed here:


